# Mussel-inspired Fluoro-Polydopamine Functionalization of Titanium Dioxide Nanowires for Polymer Nanocomposites with Significantly Enhanced Energy Storage Capability

**DOI:** 10.1038/srep43071

**Published:** 2017-02-22

**Authors:** Guanyao Wang, Xingyi Huang, Pingkai Jiang

**Affiliations:** 1 Department of Polymer Science and Engineering, Shanghai Key Laboratory of Electrical Insulation and Thermal Aging, Shanghai Jiao Tong University, Shanghai 200240, China

## Abstract

High-dielectric-constant polymer nanocomposites are demonstrated to show great promise as energy storage materials. However, the large electrical mismatch and incompatibility between nanofillers and polymer matrix usually give rise to significantly reduced breakdown strength and weak energy storage capability. Therefore, rational selection and elaborate functionalization of nanofillers to optimize the performance of polymer nanocomposites are vital. Herein, inspired by adhesive proteins in mussels, a facile modification by fluoro-polydopamine is employed to reinforce the compatibility of TiO_2_ nanowires in the fluoropolymer matrix. The loading of 2.5 vol % *f*-DOPA@TiO_2_ NWs leads to an ultrahigh discharged energy density of 11.48 J cm^−3^ at 530 MV m^−1^, more than three times of commercial biaxial-oriented polypropylene (BOPP, 3.56 J cm^−3^ at 600 MV m^−1^). A gratifying high energy density of 9.12 J cm^−3^ has also been obtained with nanofiller loading as high as 15 vol % at 360 MV m^−1^, which is nearly double to that of pure P(VDF-HFP) (4.76 J cm^−3^ at 360 MV m^−1^). This splendid energy storage capability seems to rival or exceed most of previously reported nano-TiO_2_ based nanocomposites. The methods presented here provide deep insights into the design of polymer nanocomposites for energy storage applications.

Electric energy storage plays an indispensable role in modern electronic devices and electric power systems^1^,^2^,^3^,^4^. The development of high-energy-storage-density devices is of critical importance to meet the ever-increasing urgent need. Dielectric materials, which possess the intrinsic charge-discharge capability to store and release the electrical energy through dielectric polarization and depolarization, have attracted immense interest for their potential applications in energy storage devices such as capacitors^5^,^6^,^7^,^8^,^9^,^10^,^11^. Among numerous dielectric materials, polymer nanocomposites are receiving growing concern because of the advantage combining the merits of ceramics (e.g., high dielectric constant) and polymer (e.g., high breakdown strength and ease of processing)^12^,^13^,^14^,^15^,^16^,^17^,^18^,^19^,^20^,^21^,^22^,^23^,^24^,^25^,^26^,^27^,^28^,^29^,^30^. However, the straightforward incorporation of high-dielectric-constant (high-*k*) nanofillers into low-dielectric-constant (low-*k*) polymer matrix might not be an ideal strategy for the increase of energy storage, since the large electrical mismatch between the two components might bring up an inevitable electric field distortion and the resulting reduction of breakdown strength for the nanocomposite^15^,^21^,^30^.

Development of simple and versatile strategies for modification of inorganic nanofillers has proven to be an effective method to improve their compatibility with the polymer matrices^31^,^32^. Our previous work has demonstrated that the employment of atom transfer radical polymerization (ATRP) and reversible addition-fragmentation chain transfer (RAFT) polymerization upon the modification of BaTiO_3_ nanoparticles could significantly improve their inclusion into the ferroelectric polymer matrix^33^,^34^,^35^,^36^,^37^,^38^,^39^. However, these methods intrinsically suffer from harsh experiment conditions (water-free and oxygen-free), time-consuming, and equipment-requiring drawbacks. Therefore, there is still an urgent need to develop a facile and fast method for the surface modification of nanofillers in the fabrication of polymer nanocomposites. As a burgeoning technology of self-assembled monolayers (SAMs), the dopamine self-polymerization, inspired by the composition of adhesive proteins in mussels, has gained a lot of popularity due to the one-step requirement over the last few years^32^,^40^,^41^,^42^,^43^,^44^,^45^,^46^,^47^,^48^. The thin surface-adherent polydopamine films can stick to a variety of inorganic and organic materials, by means of catechol-based adhesive compounds.

Herein, TiO_2_ nanowires (NWs) are selected rationally as the nanofillers in order to mitigate the field intensification, owing to the balanced dielectric constant of TiO_2_ with the ferroelectric polymer matrix^15^,^21^,^30^ and smaller specific surface of high-aspect-ratio nanowires compared with their nanoparticle counterparts to reduce the surface energy and alleviate the agglomeration of the nanofillers^25^,^28^. Meanwhile, we also introduce a long fluoro-chain tailed dopamine derivative, *f*-DOPA, to generate corresponding fluoro-polydopamine thin layers upon the pristine TiO_2_ NWs. The facile one-step modification guarantees the excellent inclusion of modified TiO_2_ NWs in the fluoropolymer matrix, and further facilitates the remarkable improvement of energy storage capability in comparison with pristine polymer matrix and the commercial biaxial-oriented polypropylene (BOPP). Furthermore, the elaborate functionalization of TiO_2_ NWs would also make for the reduction of temperature dependence on dielectric properties, which scarifies the reliability and performance of the dielectric materials. More striking is that high energy densities could also be achieved even at a rather low electric field, benefiting from the high loadings of attentively prepared nanofillers. We could then establish a comprehensive understanding upon the influence of aborative modification of nanofillers on the following optimization of the electric properties of polymer nanocomposites from all aspects of their properties. The results and methods presented here will provide deep insights into the design and fabrication of polymer nanocomposites for dielectric and energy storage applications at both low and high electric fields.

## Experimental Section

### Materials

Poly(vinylidene fluoride-co-hexafluoropylene) (P(VDF-HFP)) with 15% HFP was supplied by Solvay Plastics (Shanghai, China). Titanium dioxide nanopowder (TiO_2_, P25, ≥99.5%) was purchased from Sigma-Aldrich. 3,4-dihydroxy-*L*-phenylalanine (*L*-DOPA, 99%) and *p*-toluenesulfonic acid monohydrate (98%) were provided by Aladdin (China). 1*H*, 1*H*, 2*H*, 2*H*-perfluoro-1-decanol (TCI, 96%) were both purchased from Tansoole (China). Other chemicals or reagents were purchased from Sinopharm Chemical Reagent Co., Ltd. (China) and Tansoole (China).

### Synthesis of 1*H*, 1*H*, 2*H*, 2*H*-heptadecafluorodecyl 2-amino-3-(3,4-dihydroxyphenyl)propanoate (*f*-DOPA)

The synthesis of *f*-DOPA was accomplished according to previous literatures^48^. A suspension of *L*-DOPA (3.94 g, 20 mmol), 1*H*, 1*H*, 2*H*, 2*H*-perfluoro-1-decanol (9.28 g, 20 mmol), and *p*-toluenesulfonic acid monohydrate (3.80 g, 20 mmol) in toluene (100 mL) was reflux under a N_2_ atmosphere for 48 h, using a Dean-Stark trap to aezotropically remove water. After cooling to room temperature, the toluene was evaporated under vacuum. The gel-like solid residue was washed with saturated NaHCO_3_ aqueous solution and extracted with ethyl acetate. The ethyl acetate solution was further washed with brine for several times. Then the solution was dried over anhydrous MgSO_4_ for several hours and filtered. After evaporation, the raw product was dissolved in a small amount of hot ethyl acetate and crystallized from petroleum ether (60–90 °C). Yield: 8.17 g, colorless solid (63.52%).

### Surface Modification of Nanowires

The TiO_2_ NWs were synthesized by employing hydrothermal method described in previous literatures^27^. Then, the dopamine derivative *f*-DOPA was utilized to modify the surface of the pristine TiO_2_ NWs. In a typical process, 3 g TiO_2_ NWs were dispersed in 80 mL Tris-HCl buffered solution (pH = 8.5) and ultrasonicated for 1 h. Meanwhile, 4 mmol *f*-DOPA was dissolved in 40 mL 2-propanol. Then, the 2-propanol solution of *f*-DOPA was added dropwise into the aforementioned aqueous solution of TiO_2_ NWs under stirring at 60 °C. The mixture was further stirred for 48 h. With the spontaneous deposition of adherent polydopamine derivative film on the surface of nanowires, the color of the mixture was finally changed to black. After extracting from the solution with centrifugation, the nanowires were washed with deionized water and water for several times until the supernatant was nearly colorless. These surface modified nanowires were denoted as *f*-DOPA@TiO_2_ NWs.

### Fabrication of P(VDF-HFP)-based Nanocomposite Films

The typical process for the fabrication of P(VDF-HFP)-based nanocomposite films was described as follows: The functionalized TiO_2_ nanowires were first ground thoroughly and dispersed in DMF by ultrasonication for 1 h. After the addition of given amount of P(VDF-HFP), the mixture was stirred vigorously for 24 h to make it stable and homogenous. The mixture was cast into films on a glass plate with a facile scraper, and then heated at 40 °C to facilitate the evaporation of DMF. After being dried in vacuum at 40 °C for 12 h to remove the remaining trace solvent, the cast films were heated at 200 °C for several minutes and then quenched in ice water immediately. The quenched films were peeled from the glass substrates and dried at 40 °C for another 12 h. The typical thickness of these nanocomposite films is about 15 μm. Nanocomposites with different volume fractions (2.5%, 5%, 10%, and 15%) of *f*-DOPA modified TiO_2_ NWs were prepared.

### Characterization

The ^1^H, ^13^C, and ^19^F nuclear magnetic resonance (NMR) spectra were recorded on a AVANCE III HD 400 spectrometer (Bruker, USA). The morphology of the nanowires and samples was characterized with scanning electron microscopy (SEM, Nova NanoSEM 450, FEI, USA) and transmission electron microscopy (TEM, JEM-2010, JEOL, Japan). The cross-section SEM images of nanocomposite films were prepared by fracturing the films in liquid nitrogen. The samples for the TEM were prepared by dropping a few drops of the sample solution on a carbon-coated cooper grids and air-dried before measurement. The Fourier-transform infrared spectroscopy (FT-IR) of the pristine and surface modified nanowires was performed by PerkinElmer Paragon 1000 spectrometer with the range of 4000–400 cm^−1^. X-ray photoelectron spectra (XPS) of the nanowires were conducted using an Axis UltraDLD spectrometer (Shimadzu-Kratos Analytical, UK) with a monochromated Al Kα source. Thermogravimetric analysis (TGA) of nanowires was performed with NETZSCH TG209 F3 with a heating rate of 20 °C min^−1^ in a nitrogen flow (20 mL min^−1^). Both sides of the proposed nanocomposite films were sputtered by copper with diameter of 12 mm for the measurements of dielectric properties. The dielectric constant and loss tangent of the samples were measured with a Novocontrol Alpha-A high resolution dielectric analyzer (GmbH Concept 40) with the frequency range 10^−1^–10^7^ Hz at room temperature and various temperature (−50 °C–150 °C). The electric breakdown tests were carried out with a dielectric strength tester at a ramping rate of 500 V s^−1^ (Shanghai Juter High Voltage Electrical & Equipment Co., Ltd., China). All the samples used for breakdown strength have a thickness of around 15 μm. Electric displacement-electric field (*D*-*E*) loops and two probe current-voltage (I–V) measurements were conducted by a Precision Multiferroic Materials Analyzer equipped with Precision 10 kV HVI-SC and Trek MODEL 609B (Radiant Inc.). A layer of copper was evaporated on both sides of the samples to serve as electrodes (3 mm in diameter).

## Results and Discussion

### Preparation and Characterization of the Pristine and Surface Modified Nanowires

**Figure 1:**
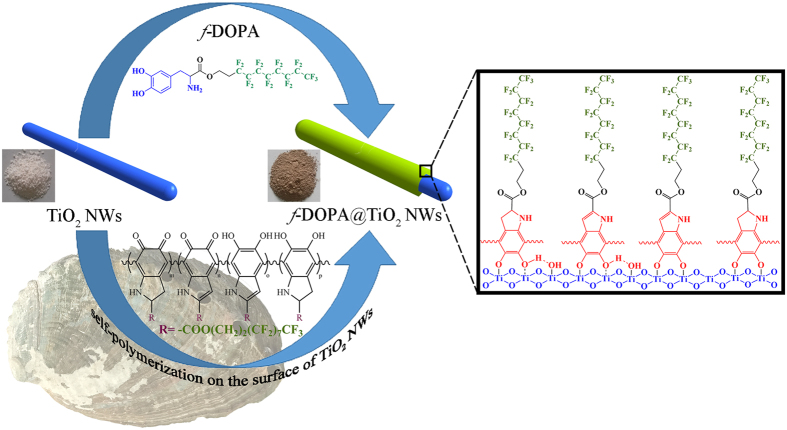
Schematic illustration of the preparation process for *f*-DOPA@TiO_2_ NWs. Inset is a photograph of a mussel.

In Fig. 1, the general synthetic route to prepare the dopamine derivative modified *f*-DOPA@TiO_2_ NWs is depicted. As shown in Fig. 1, the white TiO_2_ powders turned into brown after the modification of long fluoro-chain tailed dopamine derivative caused by the adhesion of corresponding fluoro-polydopamine layers. The structure of *f*-DOPA was confirmed by NMR spectra (See Supplementary Figs S1–S6). Compared with ^1^H NMR spectrum of *L*-DOPA (See Supplementary Fig. S1), the splitting multiplet peaks of methylene group on the catechol between 2.5 ppm and 3.0 ppm were shifted to 4.29 ppm in the ^1^H NMR spectrum of *f*-DOPA (see Supplementary Fig. S3). Besides, the multiplet peaks lined in the range of 2.50 ppm to 2.75 ppm can be ascribed to the two methylene groups of 1*H*, 1*H*, 2*H*, 2*H*-perfluoro-1-decanol. As shown in Supplementary Figs S4 and S5, the dense and weak multiplet peaks between 120 ppm and 105 ppm in the ^13^C NMR spectrum of *f*-DOPA can be attributed to the carbon signals of the long fluorocarbon chain, which were attenuated by the attached fluorine atoms in comparison with the normal carbon signals^49^. The ^19^F NMR spectrum of *f*-DOPA gave a further detailed evidence for existence of fluorine atoms (see Supplementary Fig. S6). All these results indicate that the esterification of *L*-DOPA by 1*H*, 1*H*, 2*H*, 2*H*-perfluoro-1-decanol was successful by adopting *p*-toluenesulfonic acid as the catalyst and toluene as the solvent.

**Figure 2:**
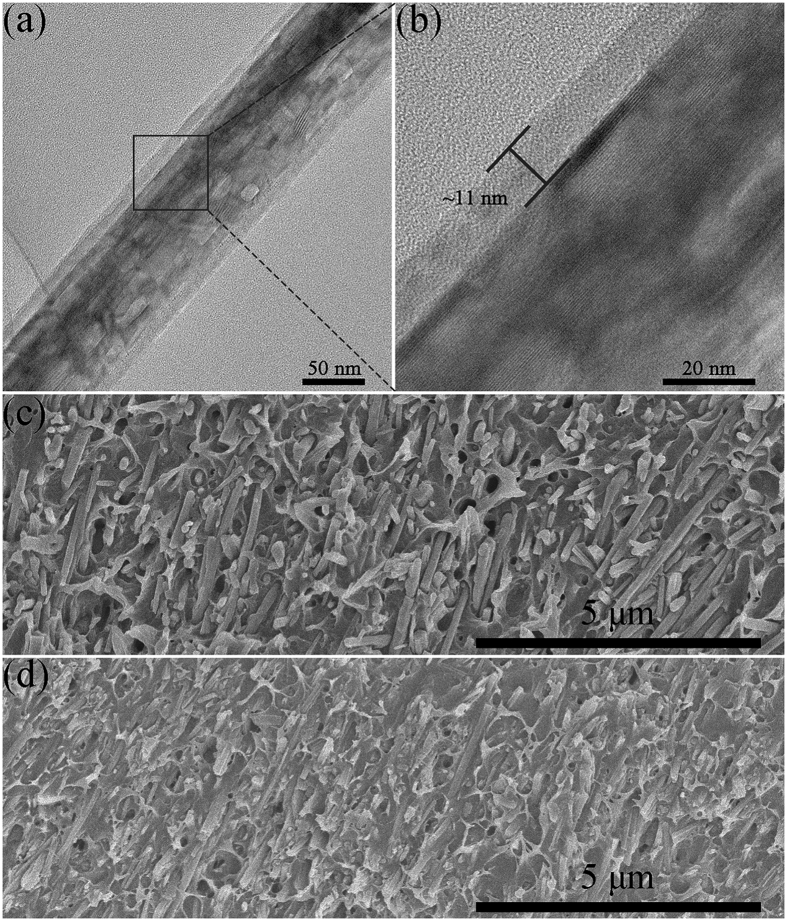
(**a,b**) TEM images of *f*-DOPA@TiO_2_ NWs. SEM images of freeze-fractured cross-section surfaces of P(VDF-HFP)-based nanocomposites with 15 vol % loading of (**c**) *f*-DOPA@TiO_2_ NWs and (**d**) TiO_2_ NWs.

SEM and TEM were carried out to characterize the morphology of nanowires and polymer nanocomposites. The free-standing TiO_2_ NWs with several micrometers in length are shown in Supplementary Fig. S7. The aspect ratio distribution of TiO_2_ NWs summarized in Supplementary Fig. S8 demonstrates that the length-diameter ratio mainly lies in the range of 19–23. As shown in Fig. 2, the distinct thin amorphous layers with ~11 nm thickness reveal the successful deposition and coating of fluoro-polydopamine on the nanowire surface, which facilitate the subsequent dispersion of nanowires in the fluoropolymer matrix. Moreover, the EDX elemental mapping images in Supplementary Fig. S9 also validate the successful attachment of proposed fluoro-polydopamine.

**Figure 3:**
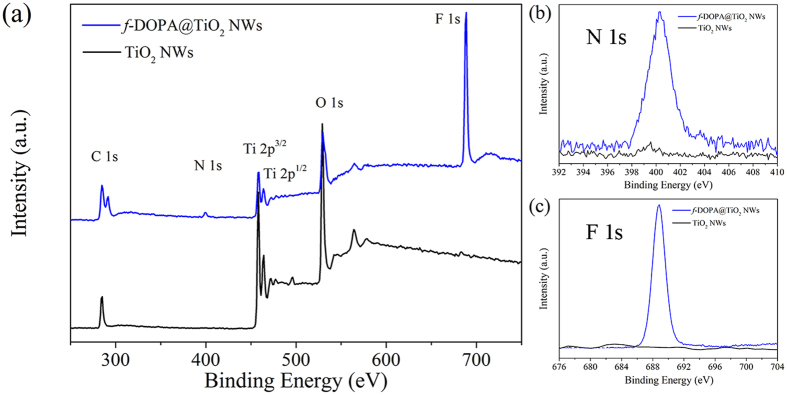
(**a**) XPS spectra of the pristine and surface modified TiO_2_ NWs. High-resolution N 1s (**b**) and F 1s (**c**) regions of TiO_2_ NWs before and after surface modification.

XPS and FT-IR are employed to further demonstrate the successful coating of fluoro-polydopamine layer on the surface of the pristine TiO_2_ NWs. As shown in Fig. 3, the presence of N 1 s peak at a binding energy of 400 eV in the XPS spectra confirmed that the nitrogen-containing dopamine derivative had been adhered on the nanowire surface through the oxidative self-polymerization of dopamine derivative. Furthermore, the apparent F 1 s peak at the binding energy of 689 eV gave a more solid evidence for the existence of fluorinated polydopamine derivative on the surface of *f*-DOPA@TiO_2_ NWs. Moreover, from the FT-IR spectra in Supplementary Fig. S10, obvious changes between 3000 and 2800 cm^−1^ can be observed after the dopamine derivatives functionalization, which can be ascribed to the C-H (methyl and methylene) stretching vibrations in the polydopamine derivatives. Meanwhile, the specific bending vibrations of C-H (methyl and methylene) are located in the range of 1500–1000 cm^−1^ for the surface modified nanowires. The intensity of the broad peak around 3500 cm^−1^ also became more remarkable after the modification in comparison with the pristine nanowires, resulting from the incorporation of amine group in *f*-DOPA.

The difference of composition and thermal stability between the pristine and surface modified nanowires was investigated by TGA. As shown in Supplementary Fig. S11, the total weight loss of the pristine TiO_2_ from 50 to 800 °C was about 0.41 wt%, indicating the successful high-temperature calcination of H_2_Ti_3_O_7_ precursor^50^. However, the removal of the water inside the precursor would inevitably bring about the defects, which benefit the following surface modification. The weight loss of *f*-DOPA@TiO_2_ NWs was notably increased to 7.68 wt%, further affirming the successful anchoring of fluoro-polydopamine layers.

### Microstructure of P(VDF-HFP)-based Nanocomposites

P(VDF-HFP)-based nanocomposite films with certain volume fractions (2.5%, 5%, 10% and 15%) of modified TiO_2_ NWs were prepared by solution blending method, respectively. As a control, Fig. 2c and Supplementary Fig. S12 present the SEM images of freeze-fractured cross-section surface of the nanocomposites with 15 vol % raw TiO_2_ NWs. Obvious agglomeration of nanowires could be observed. Besides, some nanowires were found to stretch outside the polymer matrix, indicating the weak compatibility between the pristine nanowires and polymer matrix. Such phenomenon was dramatically averted by adopting the surface modified nanowires. As shown in Fig. 2d, the fluoro-polydopamine coated nanowires are homogeneously distributed in the polymer matrix. After modification, these nanowires are buried well inside the polymer matrix. Meanwhile, the tails of these functionalized nanowires rarely stretch outside the cross-section surfaces. All these aforementioned microstructure characteristics indicate that the fluoro-polydopamine functionalized TiO_2_ NWs possess excellent compatibility with the fluoropolymer matrix.

### Dielectric Properties of the P(VDF-HFP)-based Nanocomposites

**Figure 4:**
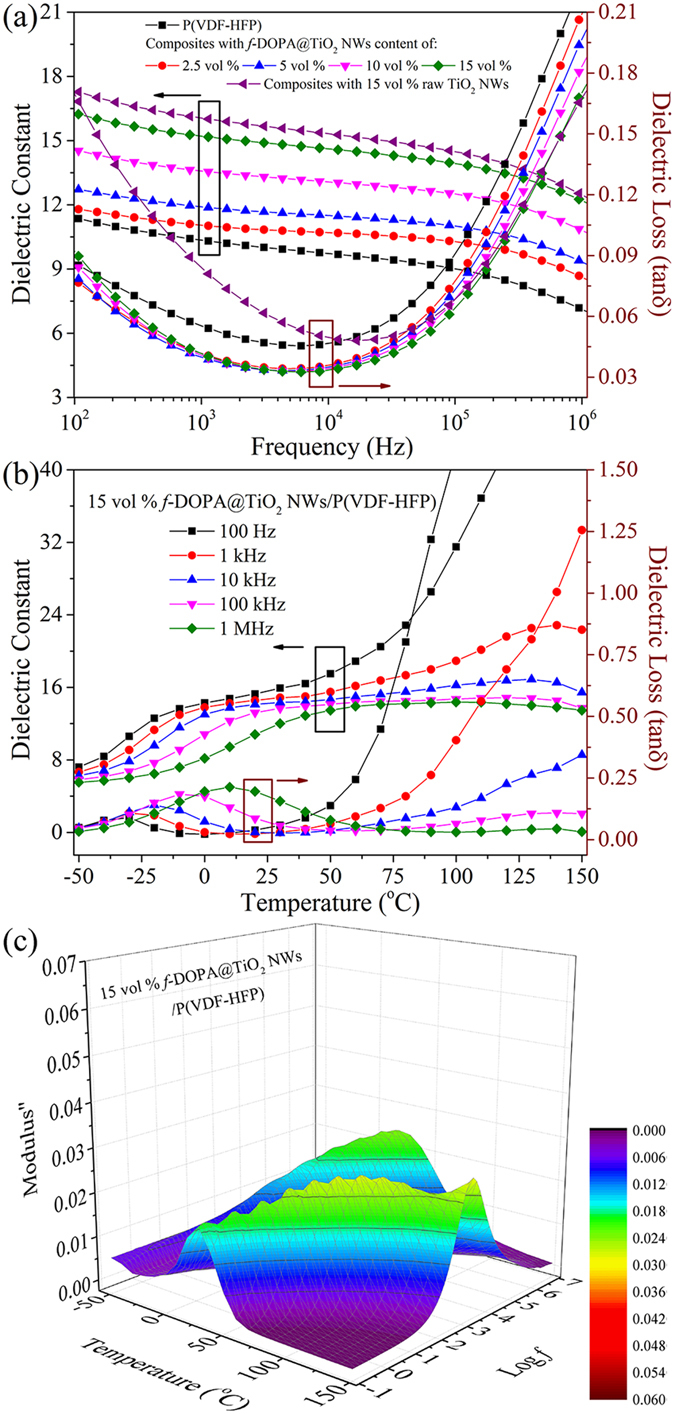
(**a**) The dielectric constant and dielectric loss (tan δ) as a function of frequency at room temperature for *f*-DOPA@TiO_2_ NWs/P(VDF-HFP) nanocomposites. (**b**) Temperature-dependent dielectric spectra of *f*-DOPA@TiO_2_ NWs/P(VDF-HFP) nanocomposites. (**c**) Frequency dependent of imaginary electric modulus at various temperature of P(VDF-HFP)-based nanocomposites with 15 vol % of *f*-DOPA@TiO_2_ NWs.

The large electrical mismatch between conventional high-*k* nanofillers (for instance, BaTiO_3_, Ba_x_Sr_1−x_TiO_3_, and PbZr_x_Ti_1−x_O_3_) and ferroelectric polymers usually gives rise to a highly distorted electric field and leads to a significantly reduced effective breakdown strength of the nanocomposites^16^,^24^,^25^,^26^,^51^. Herein, in order to maintain the high breakdown strength of polymer matrix and the flexibility of the composite films, the low volume fraction of *f*-DOPA@TiO_2_ NWs (under 15 vol %) are utilized as dopants in the ferroelectric P(VDF-HFP). The enhanced dielectric constant and restrained dielectric loss of the proposed nanocomposites over the polymer matrix are shown in Fig. 4a. Apparently, the dielectric constants of the nanocomposites possess a gradual increase with the increased loading of nanowires, resulting from higher dielectric constant of the TiO_2_ NWs relative to the polymer matrix^15^,^30^. By contrastively evaluation with the nanocomposites with 15 vol % raw TiO_2_ NWs as the reference, it can be concluded that the dielectric loss of the nanocomposites is drastically supressed, especially at the low frequencies (<10^4^ Hz), by adopting the fluoro-polydopamine modified TiO_2_ NWs. This splendid characteristic of these nanocomposites with *f*-DOPA@TiO_2_ NWs in comparison with those with pristine TiO_2_ NWs can be ascribed to the restrained interfacial polarization at low frequencies. At low frequencies, the proposed nanocomposites show slight increase of dielectric loss tangent in comparison with the pure polymer because of the interfacial polarization^33^,^52^,^53^. It should be noted that, in high frequencies, all the nanocomposites show lower dielectric loss tangent compared with the neat polymer. This phenomenon can be attributed to the enhanced interfacial interaction between the modified TiO_2_ NWs and the polymer matrix, which restricts the dipolar polarization of the P(VDF-HFP) macromolecular chains. Moreover, the dielectric loss tangents of the proposed nanocomposites show little variation in comparison with the pristine P(VDF-HFP), further implying that the surface modified TiO_2_ NWs are well dispersed in the polymer matrix.

In order to assess the dielectric performance of the proposed nanocomposites comprehensively, the dielectric properties of the nanocomposites with 15 vol % pristine TiO_2_ NWs and *f*-DOPA@TiO_2_ NWs, as well as the neat polymer, have been characterized as a function of frequency and temperature. As shown in Fig. 4b, the proposed nanocomposites exhibit typical ferroelectric behavior with the dielectric peak shifting gradually to higher temperature as frequency increases just like the pristine P(VDF-HFP) (see Supplementary Fig. S13a). Obvious broad dielectric peaks of these nanocomposites could be ascribed to the kinetics associated with the dipolar glass freezing transition^12^. The major peak at about −30 °C in the dielectric loss spectra results from the glass transition of the pure polymer. As shown in Fig. 4b and Supplementary Fig. S13b, the elaborately modified *f*-DOPA@TiO_2_ NWs show inferior elevation of dielectric constant compared with raw TiO_2_, resulting from the robust shell to hamper the role of inorganic TiO_2_ inside the host matrix. However, further investigation of dielectric loss demonstrates that *f*-DOPA@TiO_2_ NWs possess superior circumvention of interfacial polarization and restriction of movement of macromolecular chains at high temperature and low frequency.

To further explore the temperature response and temperature sensitivity of dielectric properties of the proposed nanocomposites, Fig. 4c and Supplementary Fig. S13c,d illustrate the frequency dependence of the imaginary part of the electric modulus (*M*″) of these aforementioned nanocomposites and neat polymer at various temperature, respectively. For composite systems, the interfacial polarization, also known as Maxwell-Wagner-Sillars (MWS) polarization, represents the accumulation of charge carriers at the interface of heterogeneous systems^30^,^54^. The explanation of MWS polarization would contribute to analyze the bulk relaxation behaviors of the proposed nanocomposites^30^,^55^. Two relaxation processes could be observed in these curves. The segmental motions of the amorphous region in the polymer matrix result in the relaxation peaks at high frequency^30^,^56^. The peaks occurring at low frequency and high temperature (above 50 °C) are associated with the MWS polarization of P(VDF-HFP) (see Supplementary Fig. S13c)^30^,^56^. With the increase of temperature, these peaks exhibit a gradual shift to the higher frequency, resulting from the decrease of relaxation time aroused by the enhanced mobility of charge carriers at high temperature. For those composite systems, the addition of TiO_2_ cripples the relaxation intensity of MWS polarization effectively, suggesting that the incorporation of TiO_2_ could greatly circumvent the charge aggregation at the boundaries of crystal and amorphous parts inside the polymer matrix. As discussed above, the thermo-sensitivity of the polymer nanocomposites subsides effectively upon the introduction of elaborately functionalized *f*-DOPA@TiO_2_ NWs in comparison with pristine polymer and those nanocomposites with raw nanowires.

### Energy Storage Capability of P(VDF-HFP)-based Nanocomposites

**Figure 5:**
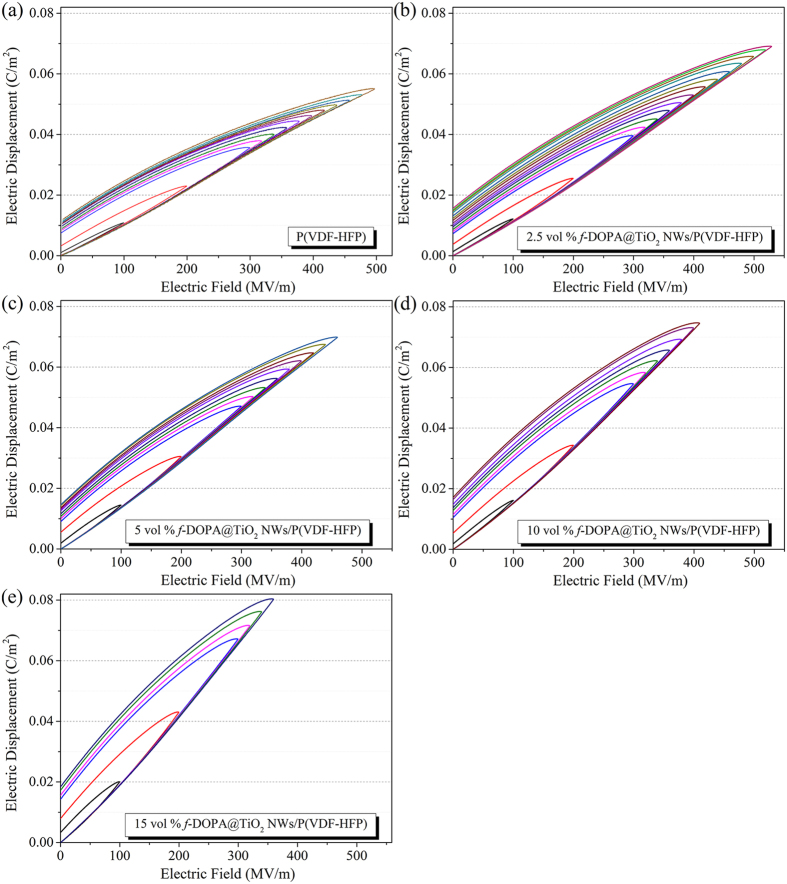
*D*-*E* loops under unipolar electric fields of 100 Hz for (**a**) pure P(VDF-HFP) and P(VDF-HFP)-based nanocomposites with (**b**) 2.5 vol %, (**c**) 5 vol %, (**d**) 10 vol %, and (**e**) 15 vol % of *f*-DOPA@TiO_2_ NWs.

**Figure 6:**
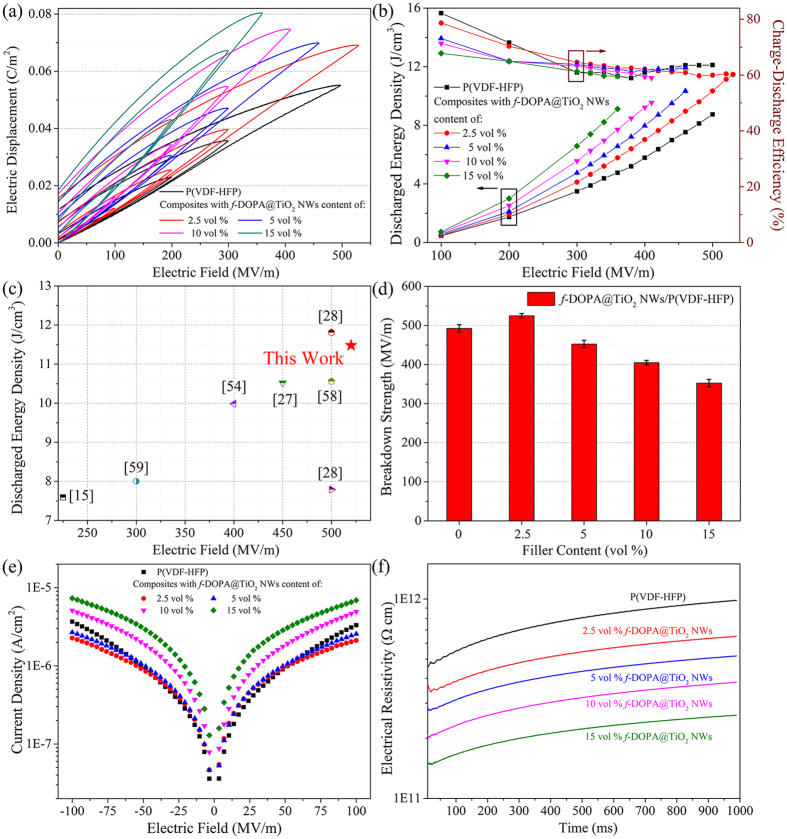
(**a**) *D*-*E* loops under unipolar electric fields of 100 Hz for *f*-DOPA@TiO_2_ NWs/P(VDF-HFP) nanocomposites with different filler concentrations. (**b**) The discharged energy densities and charge-discharge efficiency of P(VDF-HFP)-based nanocomposites with different volume fractions of *f*-DOPA@TiO_2_ NWs under varied applied fields. (**c**) Discharged energy densities of 2.5 vol % *f*-DOPA@TiO_2_ NWs/P(VDF-HFP) and other nano-TiO_2_ related polymer nanocomposites reported in previous literatures in the range of 220 MV m^−1^ to 530 MV m^−1^. (**d**) Breakdown strength of the P(VDF-HFP)-based nanocomposites at varied volume fractions of *f*-DOPA@TiO_2_ NWs. (**e**) Leakage current density of *f*-DOPA@TiO_2_ NWs/P(VDF-HFP) nanocomposites with different filler concentrations at varied applied electric field. (**f**) Electrical resistivity of P(VDF-HFP)-based nanocomposites with different volume fractions of *f*-DOPA@TiO_2_ NWs under an electric field of 100 MV m^−1^.

Generally, the energy density (*U*_e_) of a dielectric material is given by^21^,


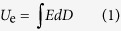


where *E* is the applied electric field, and *D* is the electric displacement. Therefore, the energy density is related to the polarization and applied electric field. Generally, the energy storage capability of the dielectric materials can be evaluated from *D*-*E* loops by a modified Sawyer-Tower circuit. Typical *D*-*E* loops of pristine P(VDF-HFP) and a series of P(VDF-HFP)-based nanocomposites with *f*-DOPA@TiO_2_ NWs are presented in Figs 5 and 6a. Obviously, relatively higher dielectric constant of the modified TiO_2_ NWs in comparison with the neat polymer matrix gives rise to the increased electric displacement for the proposed nanocomposites. Nevertheless, it can also be observed that the remnant polarizations presented in these *D*-*E* loops of these nanocomposites increase with the loading of the TiO_2_ NWs, due to the higher remnant polarization of the nanofillers than that of the polymer matrix. Typically, the high remnant polarization of the dielectric materials would result in the reduction of the discharged energy because of the decreased integrated area of *D*-*E* loops. Thus, low volume fractions of TiO_2_ NWs (under 15 vol %) were added as dopants in the fabrication of the proposed polymer nanocomposites. As demonstrated in the Fig. 5, slim *D*-*E* hysteresis loops with low remnant polarization are depicted evidently in the nanocomposites, denoting their relatively high energy efficiency discussed below.

The energy densities of the proposed nanocomposites derived from the *D*-*E* hysteresis loops and equation 1 are summarized in Fig. 6b and Supplementary Fig. S14. Obviously, the discharged energy densities of P(VDF-HFP)-based nanocomposites exhibit a gradual increase with the increasing loading of modified TiO_2_ NWs and applied electric fields, in concert with the trend of the total stored energy densities (see Supplementary Fig. S14). For example, the total stored energy densities of the nanocomposite with 2.5 vol % *f*-DOPA@TiO_2_ NWs is 19.07 J cm^−3^ at 530 MV m^−1^, much higher than that of pure P(VDF-HFP) (13.75 J cm^−3^ at 500 MV m^−1^). The nanocomposite with 2.5 vol % *f*-DOPA@TiO_2_ NWs discharges an ultrahigh energy density of 11.48 J cm^−3^ at 530 MV m^−1^, more than three times of the commercial biaxial-oriented polypropylene (BOPP, 3.56 J cm^−3^ at 600 MV m^−1^)^57^. More notably, the nanocomposite with 15 vol % nanowires also possess a striking high energy density of 9.12 J cm^−3^ at 360 MV m^−1^, nearly doubled to that of pure P(VDF-HFP) at the same electric field (4.76 J cm^−3^). In order to manifest the outstanding energy storage capabilities of these nanocomposites, the discharged energy densities of proposed nanocomposite with 2.5 vol % *f*-DOPA@TiO_2_ NWs and other previously reported polymer nanocomposites (mostly nano-TiO_2_ based) are summarized in Fig. 6c and Supplementary Fig. S15. The discharged energy density of proposed nanocomposite with 2.5 vol % *f*-DOPA@TiO_2_ at 530 MV m^−1^ seems to rival or exceed those of previously fabricated nano-TiO_2_ based nanocomposites in the range of 220–500 MV m^−1 ^15,27,28,54,58,59. These amazing results indicate their splendid potential as energy storage materials at both low and high electric fields.

For desirable energy storage capacitors, the energy storage efficiency (*η*) is also of great importance since the low energy storage efficiency can cause energy losses and reduce the lifetime of the materials. In order to maintain a high energy efficiency, one feasible approach is to suppress the dielectric loss tangent and leakage current density by improving the compatibility between the nanofillers and polymer matrix in the nanocomposites. Figure 6b shows the high efficiencies of the proposed nanocomposites by introducing dopamine derivative functionalization upon the TiO_2_ NWs. After the incorporation of modified TiO_2_ NWs, the energy efficiencies (discharged energy/total stored energy) are confronted with a gradual decrease with the increasing nanowire loadings due to the conduction loss before the approximate plateau above 300 MV m^−1 ^25,60. It’s heartening that the P(VDF-HFP)-based nanocomposites with *f*-DOPA@TiO_2_ NWs bear the efficiencies of ca. 60% at each highest tolerable electric fields, just a little lower than that of neat polymer (ca. 63.6%) at 500 MV m^−1^. This can be understood from the similar hysteresis in the polarization of the nanocomposites in comparison with that of the pristine polymer. Namely, the elaborate modification of the TiO_2_ NWs paves the way of maintenance of the high energy efficiencies, even at the maximum loading of 15 vol %. The much improved efficiencies can also be attributed to the synergistic effect of the significantly reduced leakage current densities discussed below.

Dielectric breakdown strength (*E*_b_) is of tremendous importance for the practical applications of dielectric materials. For a linear dielectric material, the equation 1 turns out to be^2^,^21^:


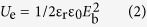


where ε_r_ is the relative dielectric constant, ε_0_ the vacuum permittivity (8.8542 × 10^−12^ F m^−1^). Therefore, the energy storage of the dielectric materials is strongly dominated by *E*_b_. Previous literatures have demonstrated that balancing dielectric constant by a rational selection of nanofiller and polymer matrix with comparable dielectric values is an effective and straightforward strategy to mitigate the field intensification effect in the ceramic/polymer nanocomposites^15^,^21^,^30^. Thus, TiO_2_ with relatively low average dielectric constant is judiciously selected as dopants in the fabrication of nanocomposite dielectrics.

Figure 6d shows the *E*_b_ of these proposed nanocomposites. As seen, *E*_b_ increases with the introduction of 2.5 vol % loading of *f*-DOPA@TiO_2_ NWs. Further increase of nanowire loading would give rise to the decrease of *E*_b_. These results can be ascribed to inevitable electric field enhancement upon the polymer matrix, resulting from the large electrical mismatch between the nanowires and the polymer matrix. Even at the highest loading of 15 vol % TiO_2_ NWs, these types of proposed nanocomposites can still withstand the electric field strength as high as ca. 350 MV m^−1^. The outstanding breakdown strength of these proposed nanocomposites might be ascribed to the following reasons: (i) The comparable dielectric constant of TiO_2_ NWs with the neat polymer and contributing surface functionalization by dopamine derivatives can effectively minimize the field distortion between the nanofillers and the polymer matrix^15^,^21^,^30^. (ii) The orientation of the high-aspect-ratio nanowires facilitates the formation of anisotropic polymer nanocomposite dielectrics, leading to anisotropy in the susceptibility of the nanocomposite films under the applied electric field and a lower concentration of electric field in the polymer matrix^29^,^61^,^62^,^63^,^64^,^65^,^66^. Furthermore, the growth of electrical treeing within the nanocomposites can be also restrained owing to the oriented nanowires, which benefit the generation of twisted pathways for treeing and scattering centers for the charge carriers^64^,^65^,^66^.

In order to further validate the influence of the leakage current within the nanocomposite films towards these aforementioned properties, the current-voltage (*I*–*V*) curves of neat polymer and the presented nanocomposites are shown in Fig. 6e. These films exhibited bipolar resistive switching properties, depending on the electric field polarity when electric field was swept from 0 to +100 MV m^−1^ and then back to −100 MV m^−1^. The leakage current densities of these nanocomposites follow a reasonable rising trend upon the increasing loading of the proposed nanowires, due to defects and voids accompanied with the incorporation of nanowires into the polymer matrix. Furthermore, the electrical resistivities shown in Fig. 6f lie in the range of 10^11^–10^12^ Ω cm, indicating their excellent insulating property qualified for the practical applications.

## Conclusions

In conclusion, mussel-inspired fluoro-dopamine (*f*-DOPA) is successfully employed to functionalize the TiO_2_ NWs. The significantly improved interface compatibility and homogeneous dispersion of modified TiO_2_ NWs in the P(VDF-HFP) matrix are guaranteed by the facile modification with fluoro-polydopamine. The dielectric constants of the present nanocomposites are confronted with a gradual increase upon the increasing loading of these modified nanowires, while the dielectric losses are still suppressed in comparison with the neat polymer. Moreover, the proposed nanocomposites can still withstand over the electric field of ca. 350 MV m^−1^. Furthermore, the energy storage capabilities of the nanocomposites are also superior to the neat polymer and some of previously reported nano-TiO_2_ based polymer nanocomposites. For instance, the discharged energy density of the polymer nanocomposites with 2.5 vol % *f*-DOPA@TiO_2_ NWs at the applied field of 530 MV m^−1^ is 11.48 J cm^−3^, more than three times of commercial BOPP (3.56 J cm^−3^ at 600 MV m^−1^). A compelling high energy density of 9.12 J cm^−3^ could be achieved at a rather low electric field of 360 MV m^−1^ even with nanofiller loading as high as 15 vol %, which is nearly doubled to that of pure P(VDF-HFP) (4.76 J cm^−3^ at 360 MV m^−1^). These results indicate that the elaborate modification of nanofillers can facilitate the subsequent performance improvement of the polymer nanocomposites. This work provides an attractive paradigm along with a unique strategy for high energy storage capability of polymer nanocomposites at both low and high electric fields.

## Additional Information

**How to cite this article:** Wang, G. *et al*. Mussel-inspired Fluoro-Polydopamine Functionalization of Titanium Dioxide Nanowires for Polymer Nanocomposites with Significantly Enhanced Energy Storage Capability. *Sci. Rep.* **7**, 43071; doi: 10.1038/srep43071 (2017).

**Publisher's note:** Springer Nature remains neutral with regard to jurisdictional claims in published maps and institutional affiliations.

## Supplementary Material

Supplementary Information
