# Supplementary material for: Mussel-inspired Fluoro-Polydopamine Functionalization of Titanium Dioxide Nanowires for Polymer Nanocomposites with Significantly Enhanced Energy Storage Capability
Source: Sci Rep. 2017 Feb 22;7:43071. doi: 10.1038/srep43071 (PMC5320529; doi:10.1038/srep43071)
Supplement: Supplementary Information [file srep43071-s1.pdf]

Mussel-inspired Fluoro-Polydopamine Functionalization of Titanium Dioxide Nanowires for Polymer Nanocomposites with Significantly Enhanced Energy Storage Capability

Guanyao Wang, Xingyi Huang*, Pingkai Jiang

Department of Polymer Science and Engineering, Shanghai Key Laboratory of Electrical Insulation and Thermal Aging, Shanghai Jiao Tong University, Shanghai 200240, China
Correspondence and request for materials should be addressed to H.X.Y. (xyhuang@sjtu.edu.cn)

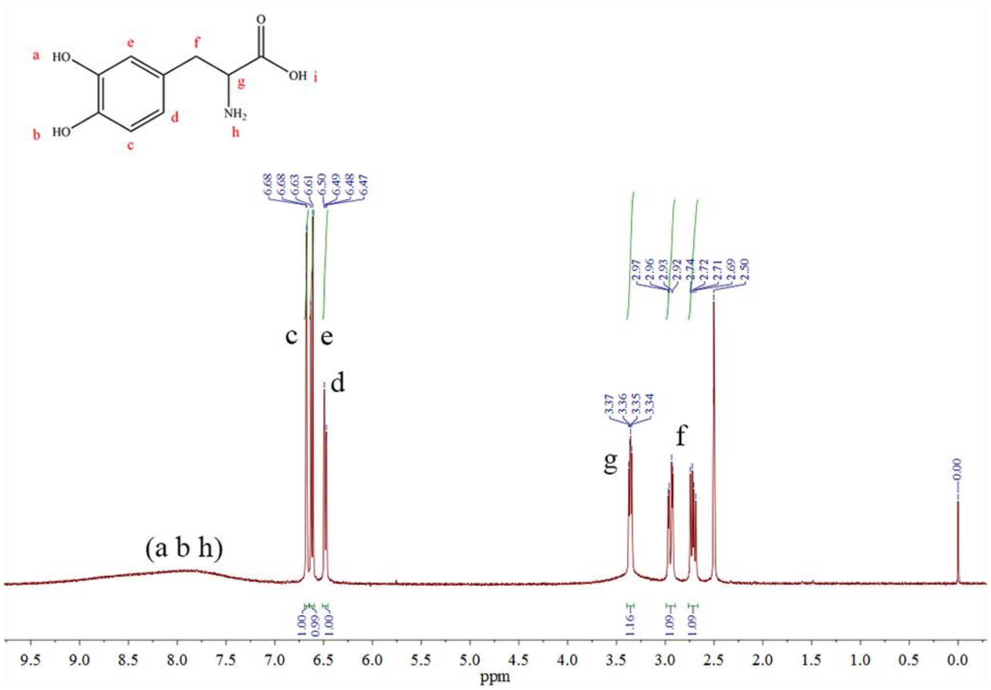

Figure S1. ^{1}H NMR spectrum of *L*-DOPA. The deuterated solvent is d_6-DMSO.

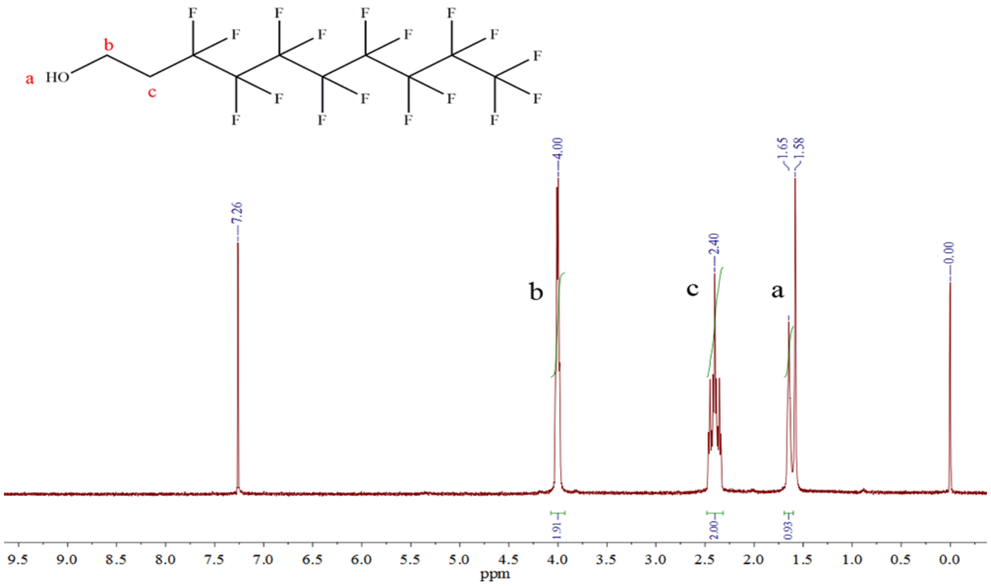

Figure S2. ^{1}H NMR spectrum of 1*H*,1*H*,2*H*,2*H*-perfluoro-1-decanol. The deuterated solvent is $CDCl_3$.

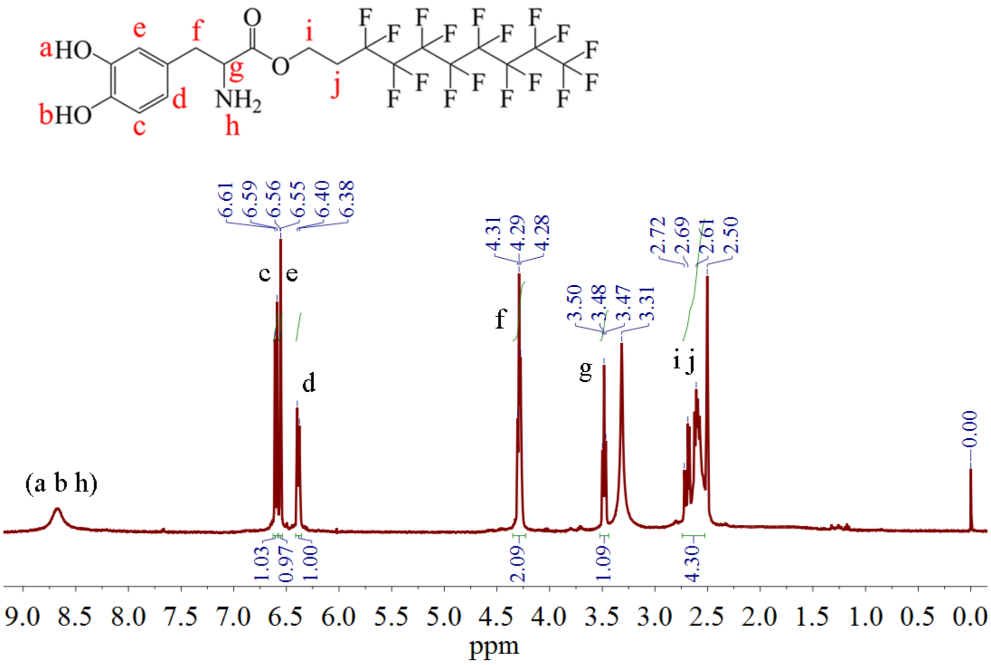

Figure S3. ^{1}H NMR spectrum of *f*-DOPA. The deuterated solvent is d_6-DMSO.

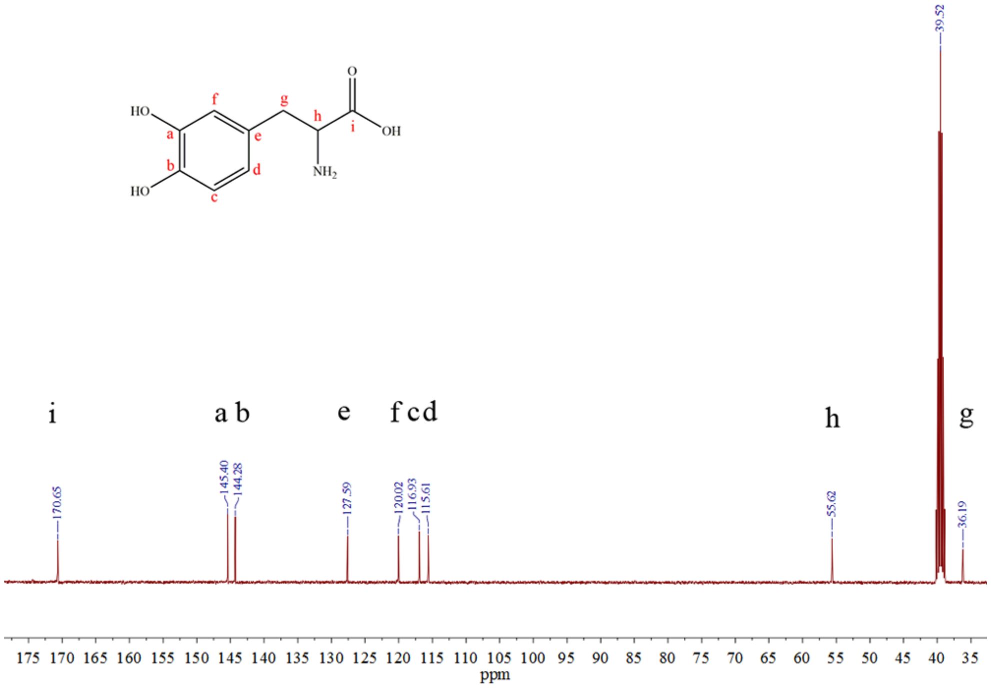

Figure S4. ^{13}C NMR spectrum of *L*-DOPA. The deuterated solvent is d_6-DMSO.

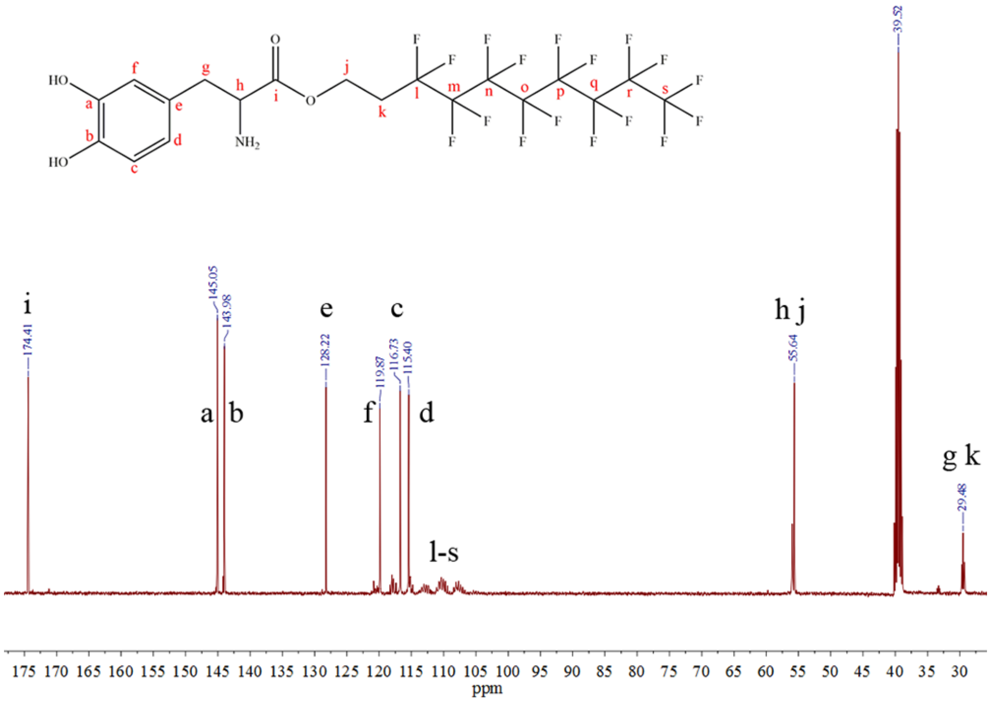

Figure S5. ^{13}C NMR spectrum of *f*-DOPA. The deuterated solvent is d_6-DMSO.

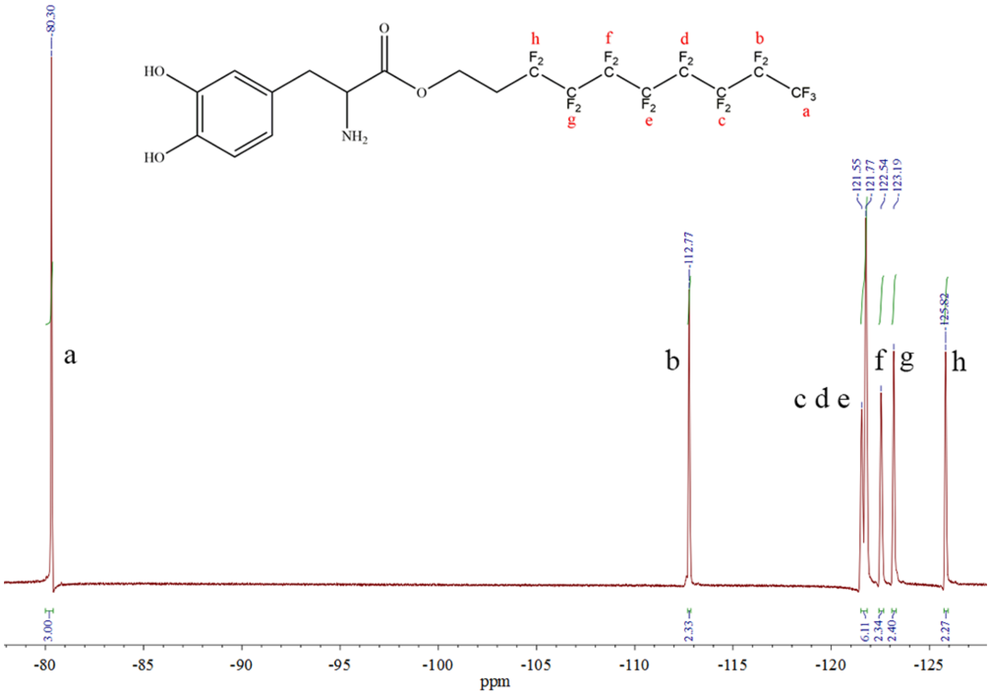

Figure S6. ^{19}F NMR spectrum of *f*-DOPA. The deuterated solvent is d_6-DMSO.

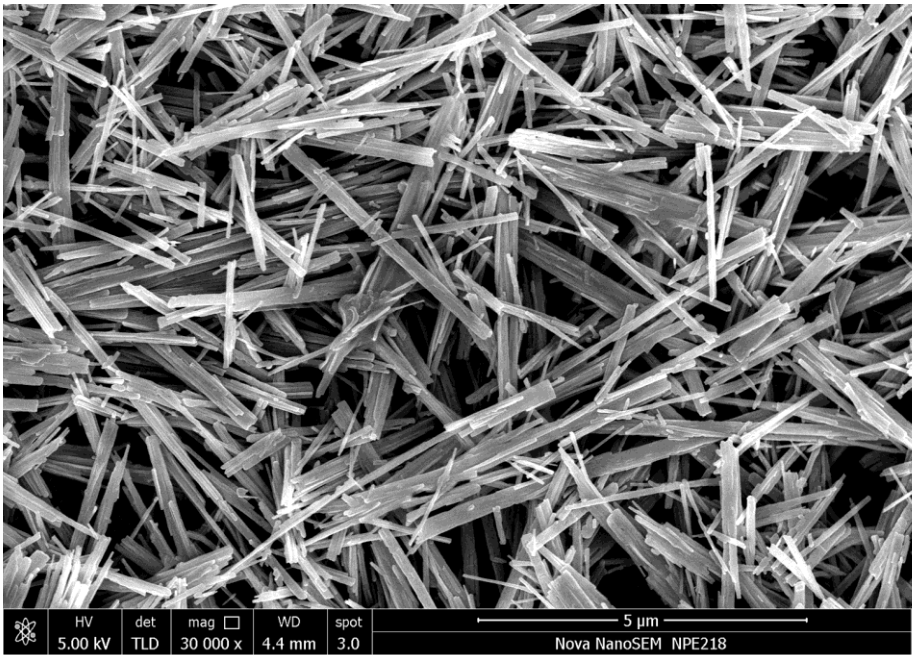

Figure S7. SEM image of the pristine TiO_2 NWs.

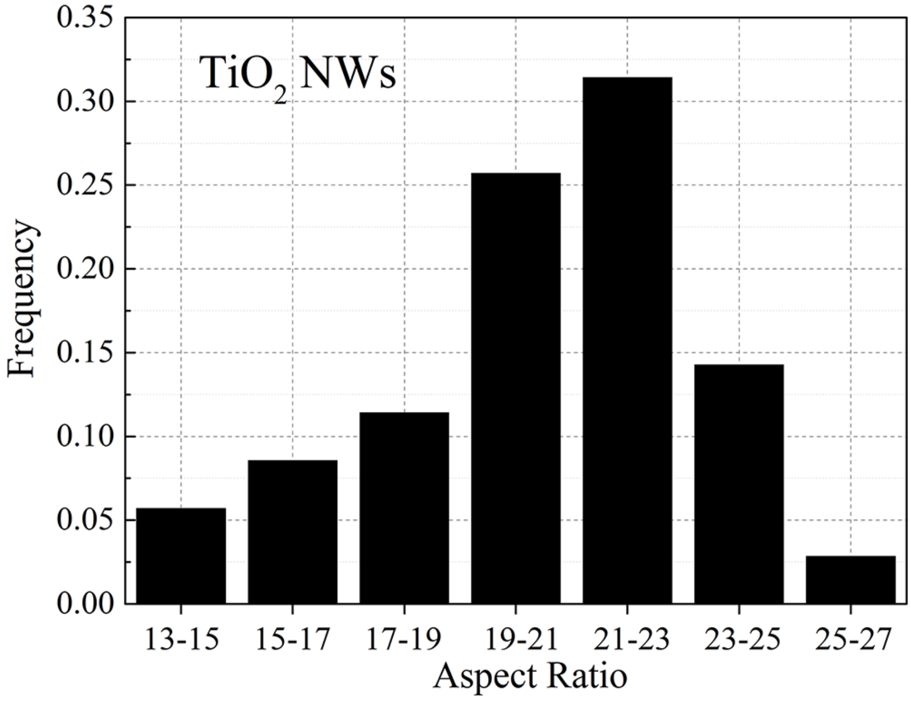

Figure S8. The aspect ratio distribution of TiO_2 NWs.

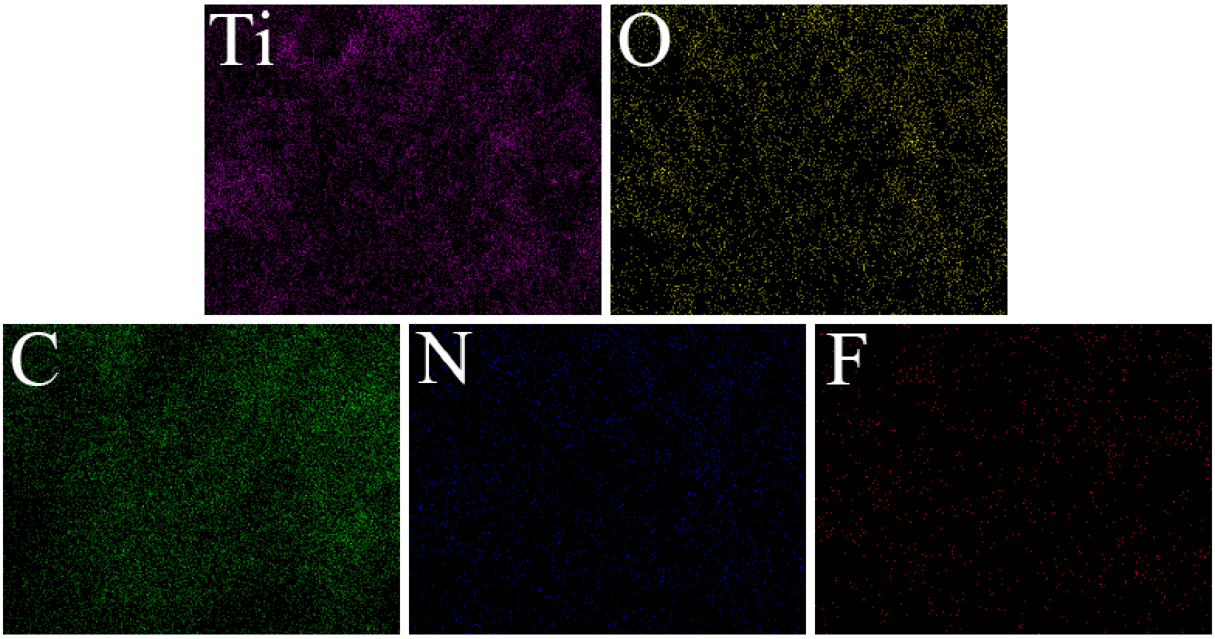

Figure S9. EDX elemental mapping images of *f*-DOPA@TiO_2 NWs: Ti mapping in cyan, O mapping in yellow, C mapping in green, N mapping in blue, and F mapping in red.

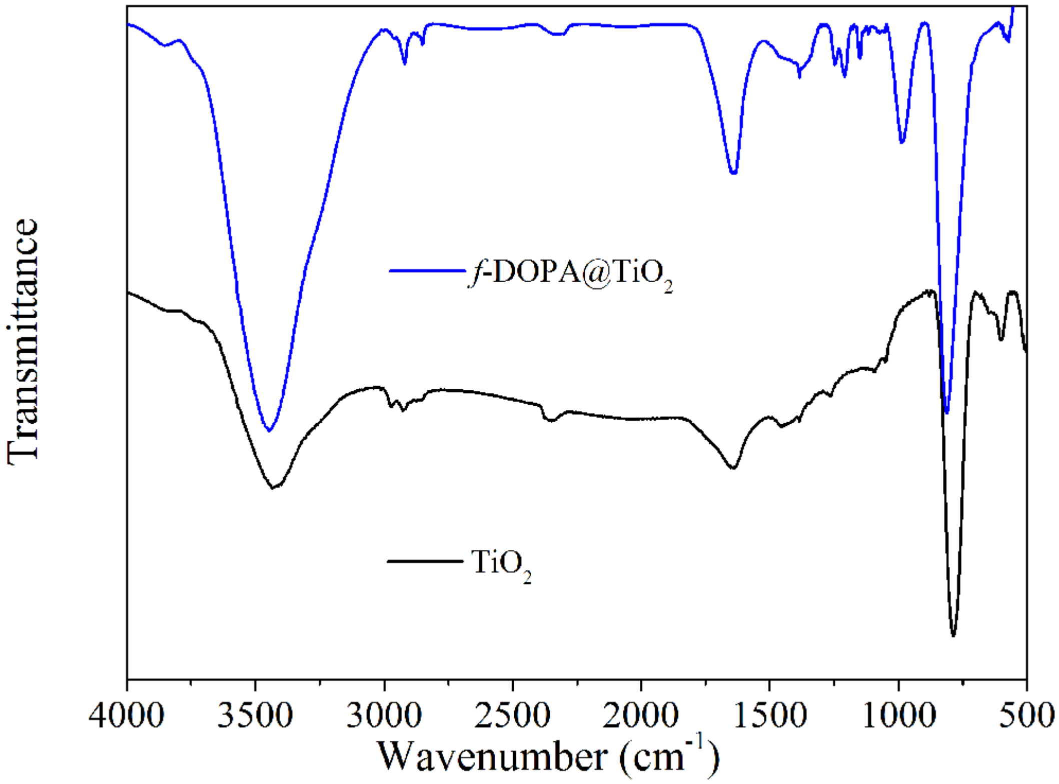

Figure S10. FT-IR spectra of TiO_2 and *f*-DOPA@TiO_2 NWs.

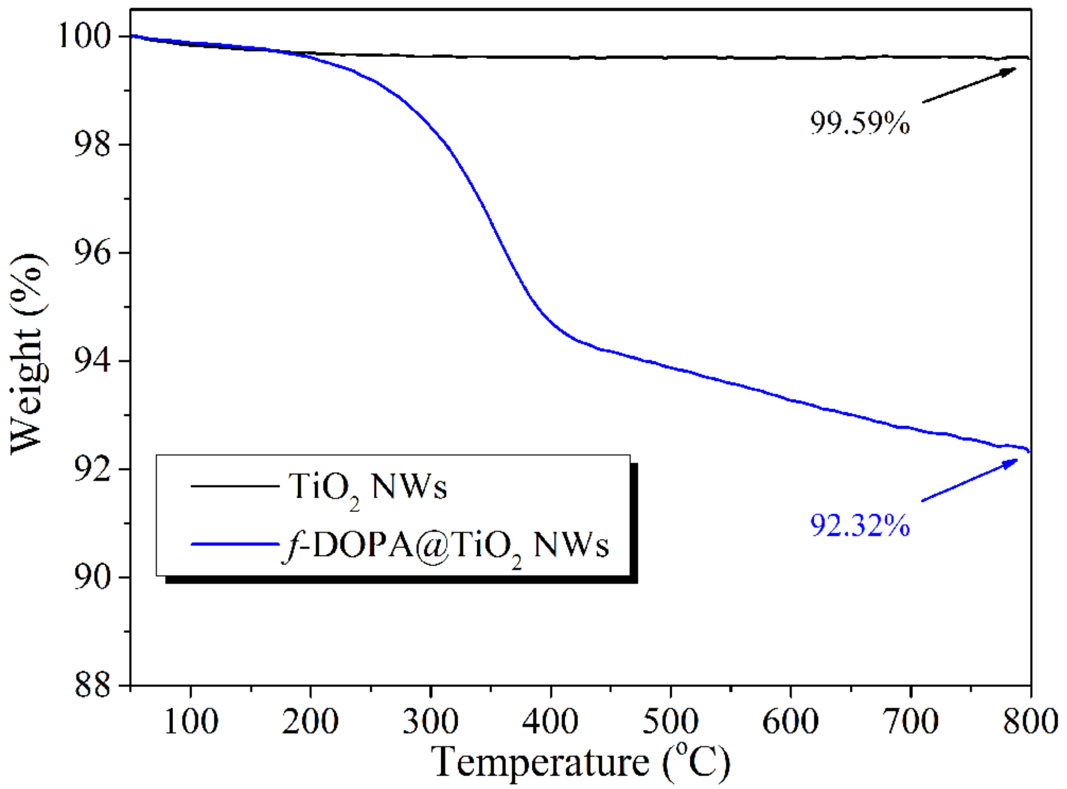

Figure S11. TGA curves for TiO_2 and *f*-DOPA@TiO_2 NWs.

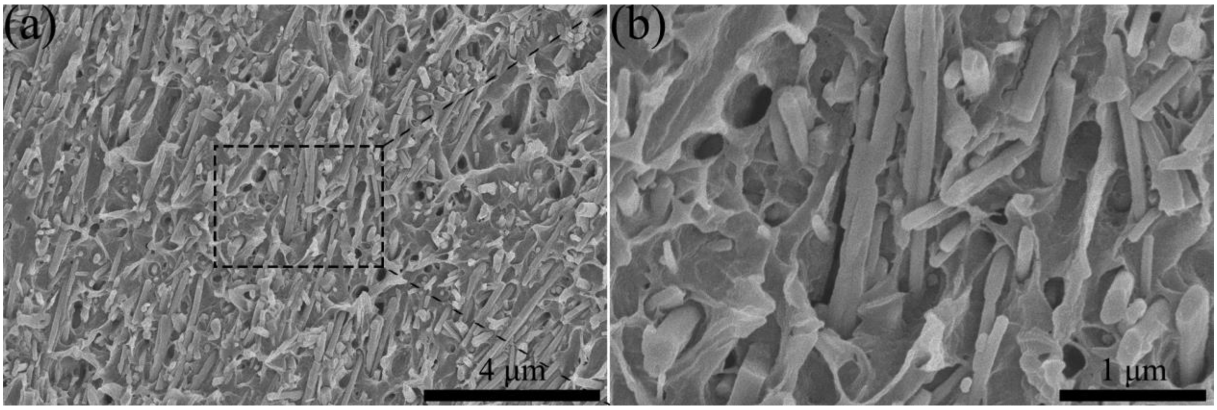

Figure S12. SEM images of freeze-fractured cross-section surfaces of TiO_2/P(VDF-HFP) nanocomposites with 15 vol % loading of the pristine TiO_2 NWs.

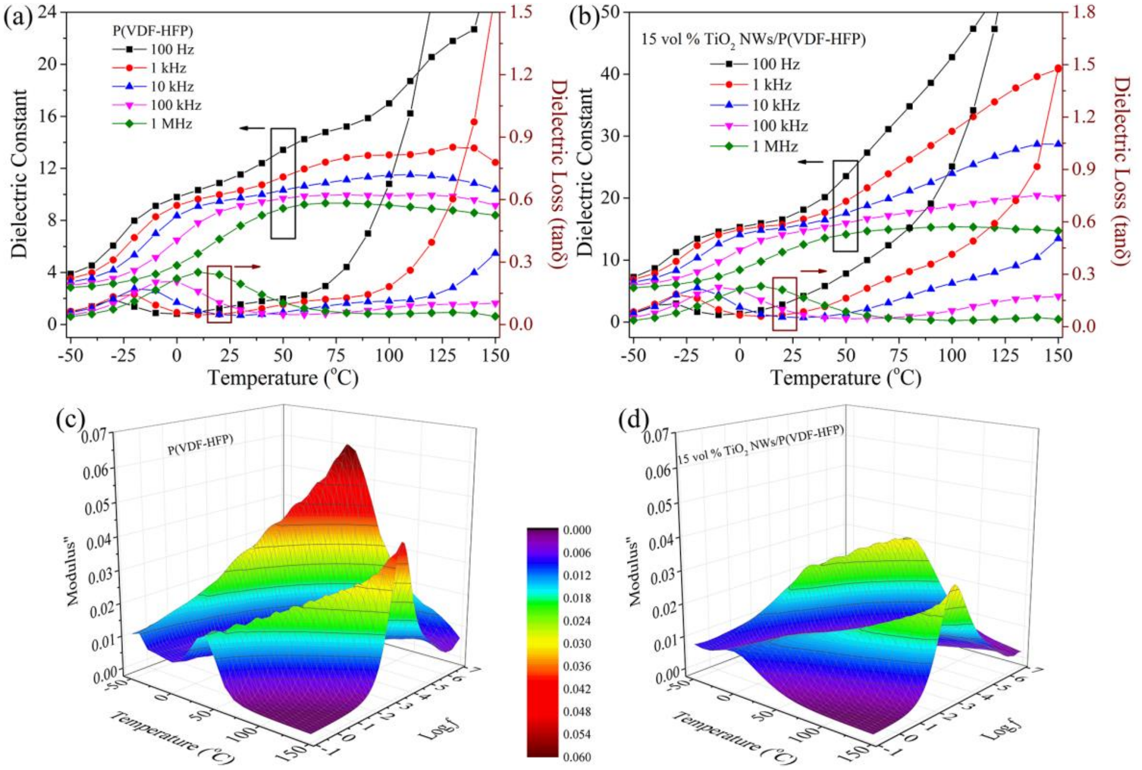

Figure S13. Temperature-dependent dielectric spectra of (a) pure P(VDF-HFP) and (b) P(VDF-HFP)-based nanocomposites with 15 vol % of pristine TiO_2 NWs. Frequency dependent of imaginary electric modulus at various temperature of (c) pure P(VDF-HFP) and (d) P(VDF-HFP)-based nanocomposites with 15 vol % of pristine TiO_2 NWs.

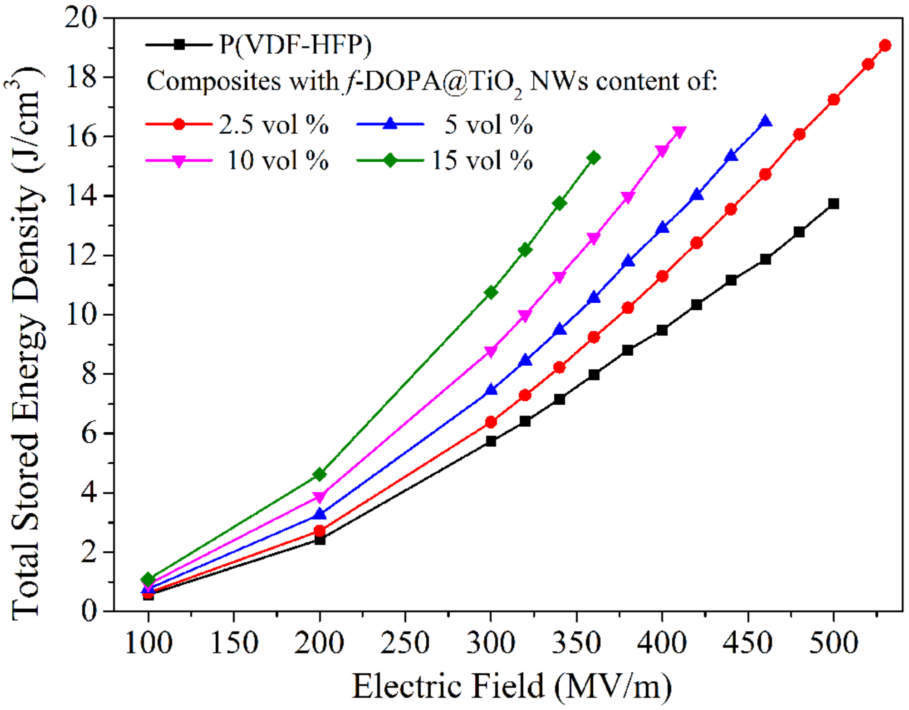

Figure S14. Total stored energy densities of P(VDF-HFP)-based nanocomposites with different volume fractions of *f*-DOPA@TiO_2 NWs under varied electric fields.

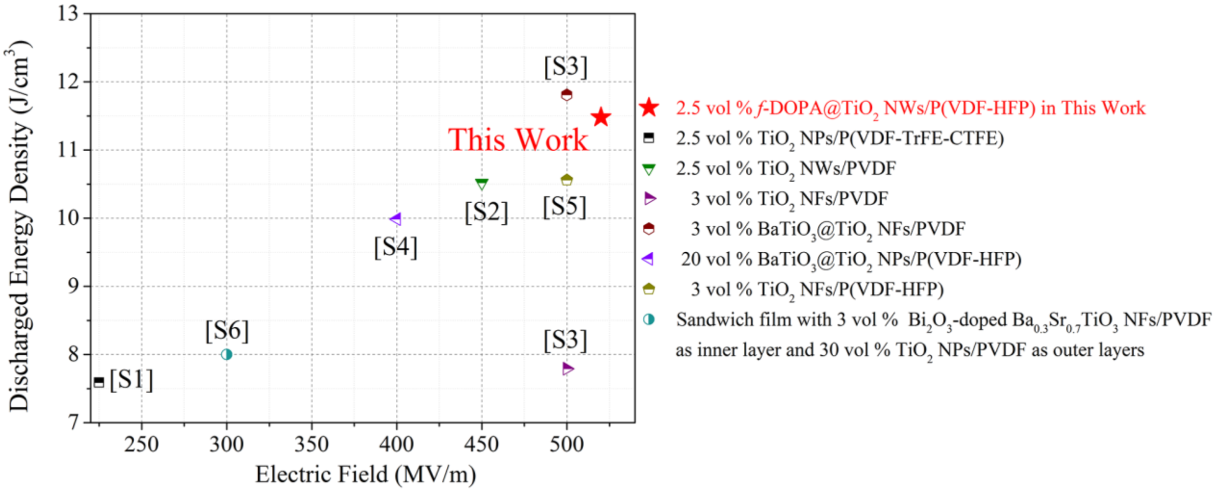

Figure S15. Discharged energy densities of 2.5 vol % *f*-DOPA@TiO_2 NWs/P(VDF-HFP) and other nano-TiO_2 related polymer nanocomposites reported in previous literatures in the range of 220 MV m^{-1} to 530 MV m^{-1} [S1-S6].

References

S1 Li, J. *et al.* Nanocomposites of ferroelectric polymers with TiO_2 nanoparticles exhibiting significantly enhanced electrical energy density. *Adv. Mater.* **21**, 217-221, (2009).

S2 Tang, H. & Sodano, H. A. High energy density nanocomposite capacitors using non-ferroelectric nanowires. *Appl. Phys. Lett.* **102**, 063901/1-063901/4, (2013).

S3 Zhang, X. *et al.* Ultrahigh energy density of polymer nanocomposites containing $BaTiO_3$@TiO_2 nanofibers by atomic-scale interface engineering. *Adv. Mater.* **27**, 819-824, (2015).

S4 Rahimabady, M., Mirshekarloo, M. S., Yao, K. & Lu, L. Dielectric behaviors and high energy storage density of nanocomposites with core-shell $BaTiO_3$@TiO_2 in poly(vinylidene fluoride-hexafluoropropylene). *Phys. Chem. Chem. Phys.* **15**, 16242-16248, (2013).

S5 Zhang, X. *et al.* Giant energy density and improved discharge efficiency of solution-processed polymer nanocomposites for dielectric energy storage. *Adv. Mater.* **28**, 2055-2061, (2016).

S6 Hu, P. *et al.* Highly enhanced energy density induced by hetero-interface in sandwich-structured polymer nanocomposites. *J. Mater. Chem. A* **1**, 12321-12326, (2013).
